# The Effect of Cochlear Size on Cochlear Implantation Outcomes

**DOI:** 10.1155/2019/5849871

**Published:** 2019-06-04

**Authors:** Jafri Kuthubutheen, Amandeep Grewal, Sean Symons, Julian Nedzelski, David Shipp, Vincent Lin, Joseph Chen

**Affiliations:** ^1^Department of Otolaryngology Head and Neck Surgery, Fiona Stanley Hospital, Perth, Western Australia 6150, Australia; ^2^Sunnybrook Health Sciences Centre, Toronto M4N3M5, Canada; ^3^University of Toronto, Toronto M5S 1A1, Canada

## Abstract

**Objectives:**

To determine if cochlear duct length and cochlear basal diameter, measured using routinely available radiology software, affect hearing outcomes after cochlear implantation with two different length electrodes.

**Methods:**

55 patients who received a Med-El Flex electrode were retrospectively reviewed. 34 patients received the Flex 31 electrode (31mm) and 21 patients received the Flex 28 electrode (28mm). Preoperative high-resolution CT scans of the temporal bone were reformatted in the axial and coronal plane. The basal diameter of the cochlear (A-value) and the outer-wall lengths of the cochlear duct were measured using readily available imaging software. Postoperative plane X-rays were used to determine the degree of electrode insertion and the number of electrodes within the cochlea and speech discrimination scores at 6 months were evaluated.

**Results:**

The cochlear metrics obtained were comparable with those previously published in the literature. There was no significant difference in the degree of insertion or speech outcomes between the two electrode lengths. However, when the group who had received the shorter electrode were analysed, there was an association seen between both cochlear duct length and cochlear diameter and speech outcomes.

**Conclusions:**

Cochlear size may be a factor in determining speech outcomes that cannot be explained solely by insertion depth or degrees of insertion. Further studies are required to determine if cochlear duct length is an independent predictor of speech outcomes.

## 1. Introduction

Hearing preservation cochlear implantation (CI) assumes atraumatic electrode insertion [1]. Hearing preservation has been achievable through factors such as the use of flexible and slim electrodes, steroids, and “soft” surgical techniques [2–4]. There is a well-recognized variability in cochlear duct lengths (CDL) between individuals [5]. This inevitably results in cochleae that are more or less suited to the electrode being chosen because currently a single electrode length is commonly chosen for the majority of ears being implanted. Hearing outcomes may therefore be affected by the anatomical characteristics of the cochlea. The temporal bone CT scan is often used routinely to assess the preoperative anatomical status prior to cochlear implantation although specific cochlear metrics are not always assessed prior to surgery.

The aim of this study was to determine if cochlea metrics can be reliably obtained using routine imaging software and whether cochlear duct length and cochlear diameter were a factor in determining hearing outcomes for two different length electrodes.

## 2. Materials and Methods

The study was conducted at a tertiary adult implant center with ethics approval. A retrospective chart review of patients receiving a cochlear implant over a 5 year period was conducted. A total of 55 postlingual deafened adults were included. CI was offered after a failed trial of hearing aids and a best aided HINT sentence score in quiet of less than 60% [6]. Patients had a range of residual low frequency hearing with moderately severe to profound high frequency loss above 4000Hz outside the criteria for electric-acoustic stimulation.

Preoperative testing included a pure tone average (PTA) at 250Hz, 500Hz and 1000Hz, and word discrimination with CNC monosyllabic word testing in quiet at 60dB SPL [7]. The Flexsoft™ (Flex 31) electrode was the standard electrode at our centre from 2008 to 2012 followed by the Flex28™ (Flex 28) electrode thereafter. A change in preoperative assessment with the shorter electrode meant that, apart from the HINT and CNC word testing, the Flex 28 group also had preoperative AZBio sentence testing at 60dB SPL [8].

Three surgeons performed surgery. A routine postauricular approach with facial nerve monitoring was used. A single dose of 8mg of dexamethasone was given on induction. A cortical mastoidectomy was performed followed by a posterior tympanotomy through the facial recess. The round window niche was lowered and after bone dust was removed and the round window membrane was opened and the electrode inserted gradually until full insertion or resistance. Insertion was performed using a combination of freehanded or instrument guided (using surgical claw, micro angled forceps or jeweller's forceps). A small soft tissue plug was placed in the niche, with the remainder of the electrode coiled in the mastoid cavity before closure.

The Flex 31 electrode measures 31.5mm with 19 platinum electrode contacts spaced over 26.4mm. The diameter at the basal end is 1.3mm and the tip measures 0.5mm x 0.4mm. The Flex 28 electrode measures 28mm with 19 platinum electrode contacts spread over a shorter distance of 23.1mm. The diameter at the basal end is smaller at 0.8mm but the tip dimensions are the same as the longer electrode [9].

Only patients who had a preoperative temporal bone CT scan performed at our centre were included. The temporal bone CT scan was performed on a GE Lightspeed Plus 64 multi-slice CT scanner. The axial images were 0.625mm in thickness and the oblique-coronal plane images were 0.6mm in thickness. Image processing and measurements were performed on a GE AW-workstation release 4.4 running the Volume Viewer software version 8.3.65.

**Figure 1:**
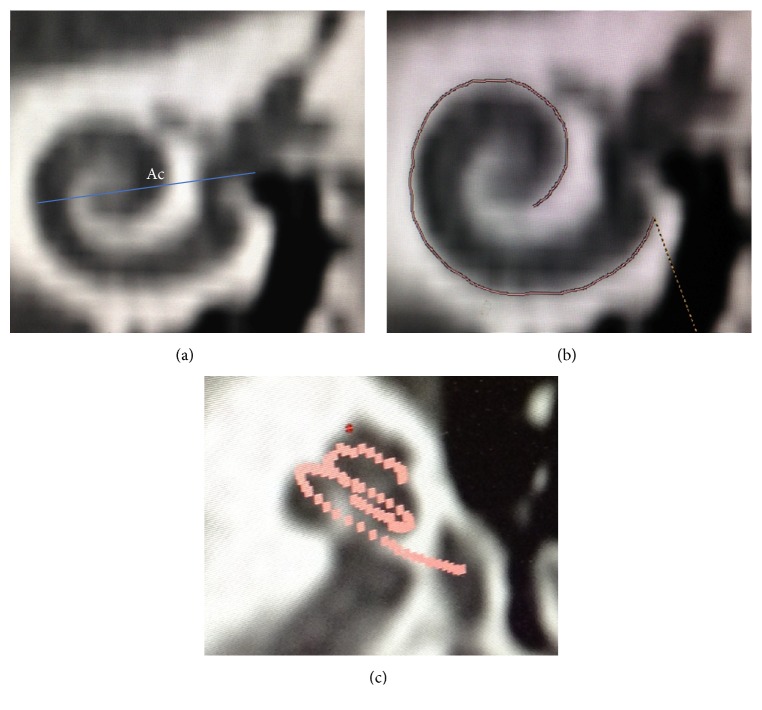
The A value measured in the oblique coronal plane (a). A straight line-measuring tool (b) is used to measure the outer wall cochlear duct length to 360 degrees. A side profile view (c) indicates the individual points used to calculate the length till 720 degrees.

The cochlea was reformatted in the oblique coronal plane to obtain the entire basal turn in a single view. The straight measuring tool was then used again to measure the distance from the most lateral bony wall, through the modiolus, to the interface between air and soft tissue at the round window midpoint. This was termed the A-value measurement as per Escude (Figure 1(a)) [10].

The cochlea was then centered about its modiolus so that by scrolling superiorly the cochlear turns were gradually brought into view. A curved measuring tool was then used to measure the distance starting at the lateral wall of the round window (most proximal portion of the basal turn of the cochlea) and followed until 360 degrees of rotation was reached (Figure 1(c)). The lateral wall was then progressively traced to 720 degrees. An axial view of the distance traced was then visualised to confirm the measurement (Figure 1(c)). The degrees of electrode rotation were measured relative to the line used to measure the Ac value and a line perpendicular to this, centered on the modiolus in a method similar to Erixon [11]. Measurements were made to 720 degrees because there was poor resolution approaching the helicotrema.

Postoperatively, CNC word scores were measured at 6 months in all subjects. In the Flex 31 group, the HINT sentence test was performed at 6 months, and in the Flex 28 group the AZBio sentence test in quiet was also performed at 6 months. Postoperative plain X-rays of the skull were performed within 24 hours of after surgery using modified Stenver's view. A senior radiologist blinded to the electrode type viewed the images. The degree of electrode insertion and the number of electrodes within the cochlear were reported [12]. This method used is considered to be better than linear insertion depth because it is independent of the distance from the electrode to the modiolus [13]. Statistical analysis was performed using SPSS version 13.0 for windows. Comparison between two independent groups was performed using the Mann-Whitney U test and Chi square analysis with a significance level of less than 0.05 being considered as statistically significant. Linear regression analysis was performed to determine the correlation between data sets.

## 3. Results

There were 34 patients implanted with the Flex 31 and 21 patients implanted with the Flex 28 electrode. The mean age for both groups was 62 years and 63 years of age, respectively, with no significant difference the two groups. The mean age for the entire cohort of patients was 62 years (SD 12.8 years). There were 30 male and 25 female patients and 24 left and 31 right ears implanted. The etiologies of hearing loss were similar between the two groups with the majority of hearing loss due to idiopathic progressive sensorineural hearing loss (Table 1).

The preoperative speech and PTA measures are shown in Table 2. The preoperative PTA was similar between the two groups at 69dB for the Flex 31 group and 72dB for the Flex 28 group. The preoperative HINT score was higher for the Flex 28 group (mean of 45.2%) compared to the Flex 31 group (36%) although this was not statistically different. However, the preoperative best-aided CNC word score was significantly higher in the Flex 28 group (30.9%) compared to the Flex 31 group (12.3%, p<0.05). This may reflect widening of the criteria in the more recently implanted group to include patients with better speech discrimination scores. The preoperative AZBio sentence score in the Flex 28 group was 33.3% (SD 20.6). A corresponding score was not available for the Flex 31 group because this was not part of the routine implant workup at the time.

The postoperative CNC word scores at 6 months were comparable between the two groups at 52.6% (Flex 31) and 59.7% (Flex 28) (Table 2). Taking into account the preoperative CNC word scores, the CNC score shift (difference between the preoperative and the postoperative word scores) was also similar between the two groups.

**Figure 2:**
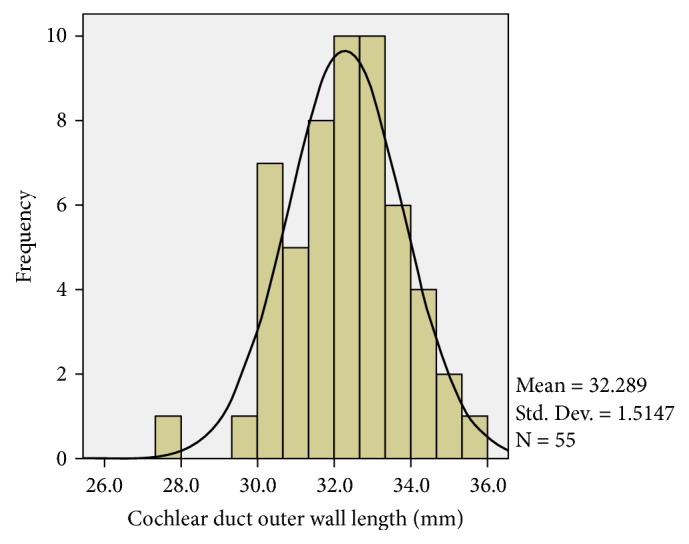
A histogram showing the normal distribution of the outer wall CDL to 720 degrees in all 55 patients.

The results of the different cochlear metrics are shown in Table 3. The A-values varied between 8.1 and 9.8mm. The outer wall CDL to 720 degrees ranged from 27.8mm to 35.9mm with a mean of 32.3mm (SD 1.51). The mean basal turn outer wall length to 360 degrees was 21.3mm (SD 1.0mm) or 65.9% of the length to 720 degrees. Plotted outer wall lengths approximate a normal distribution (Figure 2). There were no statistically significant differences in the cochlear metrics between the two electrode groups, indicating that the electrodes were implanted into groups of patients with similar cochlear morphologies.

**Figure 3:**
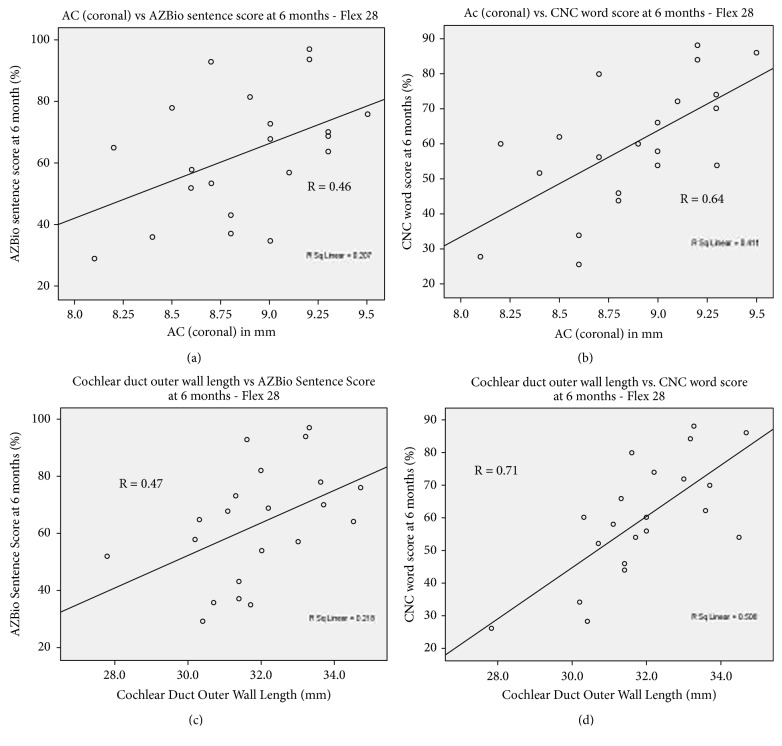
A plot showing a statistically significant correlation between the Ac value and the AZBio sentence score (a) and CNC word score (b) in the Flex 28 group as well as the correlation between the outer wall cochlear duct length and the AZBio sentence score (c) and CNC word score (d) at 6 months.

In the Flex 28 group, the A-value was correlated with the postoperative CNC word score (R=0.64) and the AZBIO score(R=0.46). In other words, when the shorter electrode was used, a larger basal diameter was associated with better speech discrimination (Table 4, Figure 3). These associations are not seen in the group who had the longer Flex 31 electrode. In the Flex 28 group, the outer wall CDL measurements were also significantly correlated with CNC word and AZBIO sentence scores (Table 4, Figure 3). The strongest correlation exists for the CNC word score (R=0.71). In the Flex 31 group, this association is not seen. Therefore, when a shorter electrode was used, longer CDLs are associated with better postoperative speech performance.

### 3.1. Degree of Insertion and Number of Channels Inserted

In both groups, the degree of insertion or the number of channels inserted did not correlate with speech outcomes at 6 months. The degree of electrode insertion and number of electrodes inserted was higher in the Flex 28 group (525 degrees and 11.3 channels) compared to the Flex 31 group (489.4 degrees and 10.9 channels) although this was not significantly different. For both groups, the number of channels inserted on plain X-ray was positively correlated with the degrees of electrode insertion, with a stronger correlation for the Flex 28 group (R=0.71 vs. R=0.589). In other words, the greater the degree of electrode insertion the greater the number of channels visualized to have been inserted on plain X-ray. In the Flex 28 group and the Flex 31 group, neither the degree of insertion nor the number of channels inserted was correlated with any cochlear measurements.

## 4. Discussion

Previous cadaveric studies have shown that CDLs and the number of cochlear turns varies between individuals [5, 14]. The normal distribution of our data for CDL to 720 degrees is consistent with this and other studies [5, 15].

The technique used to measure the CDL and the A-value was chosen for its ready availability. All our cochlear implant candidates now have standardized temporal bone CT scans as part of their workup and no specialized software is required to process the images unlike other automated techniques [16]. However, there are some limitations of this technique. This method has not been validated with histopathology, micro CT, or high-tesla MRI and may under- or overestimate the true CDL. There is also difficulty in determining the CDL beyond 720 degrees near the helicotrema where the resolution is poor. In addition, the A-value is difficult to measure from a standard CT sequence without having to perform some reformatting of the images, step which is possible to do with some practice as long as the images are iso-volumetrically acquired. However, our measurements are comparable with previously published studies.

The plain postoperative X-ray was used to determine angular insertion [12]. However, determining the number of electrodes in the cochlea can be subjective. A postoperative CT scan is more accurate albeit with higher radiation doses. Differences in speech testing protocols also limited some comparisons. In addition, we did not control for factors such as duration of deafness and patient compliance. It remains to be seen whether the correlations found extend beyond 6 months.

When comparing CDLs across different studies, a standardized method needs to be utilized in order to make meaningful comparisons, a need which has been echoed by other authors [17]. The outer wall of the cochlea margin for measuring the CDL is a common technique but due to interpatient variability, measurements of the CDL vary across the literature [10, 11, 15, 18–20]. The largest study of its kind to date with data from 436 cochleae in 218 patients using cone beam CT has found a mean CDL of 37.9mm with a range from 30.8mm to 43.2mm [15].

In comparison to these studies, our study measured the outer wall CDL to 720 degrees across 54 patients and found a mean length of 32.29mm. The human cochlea may vary from 774 to 1037 degrees, with a mean of 929 degrees [11]. With this correction, our outer wall CDL measurement of 32.29mm translates to mean, corrected total outer wall CDL of 41.7mm (range 34.7mm to 46.5mm), which compares very well with the available literature.

Our mean basal diameter value of 8.91mm is slightly longer than Martinez-Monedero's value of 8.39mm (SD 0.76) and Ketten's value of 7.91mm (although the central fluid space was used as the reference in the latter) but slightly shorter than Escude's value of 9.23mm (SD 0.53) and Connor's value of 9.36mm (SD 0.31) [10, 14, 21, 22]. Our standard deviation was similar to these studies and suggests our measurements are comparable to previously published studies.

In our study, the basal turn represented 65.9% of the CDL to 720 degrees. This is comparable to Hardy's figure of 57.9%, Escude's figure of 59%, and Erixon and Rask-Andersen's figure of 53%* of the total* CDL [5, 10, 18]. Our absolute basal turn length to 360 degrees measurement of 21.3mm compares favorably with Erixon and Rask-Andersen's figure of 22.6mm, Escude's figure of between 20 and 25mm but is slightly longer than Hardy's figure of 18.23mm.

Our insertion angles with the Flex 28 are similar to that obtained by Franke-Trieger (Franke-Trieger et al., 2013). However, our results indicate a lower insertion angle for the Flex 31 electrode compared to previously published figures which also vary considerably. Franke-Trieger in the same 10 adult temporal bones found a mean insertion angle of 673 degrees. However, in Trieger's paper, complete insertion was not achievable in all subjects with the 31mm electrode [23]. Hamzavi in 10 patients implanted obtained insertion degrees of over 500 degrees, with a mean of 542 degrees. However, in their study, a cochleostomy was used [24]. Boyd showed a mean angular insertion of 630 degrees in 85 patients [25]. Ibrahim in a temporal bone study found an insertion depth of 610 degrees [26].

We found only two studies with lower insertion angles than our study for the Flex 31 electrode. The first was a study by Radeloff using a Med-El Combi 40+ electrode (31.5mm) via a cochleostomy in a 28 temporal bones [27]. However this study found higher insertion angles when full insertion was achieved but resulted in more traumatic scala vestibuli insertions. Mick et al. examined the insertion depths of 49 patients with a Flex 31 electrode, of which 31 patients were included in our study. The mean insertion depth was 468.5 degrees, which was slightly lower than our figure but included both round window and cochleostomy insertions [28].

Our study found no correlations between cochlear size and the degree of insertion. This finding is unlike previously reported by two studies [10, 23]. Escude examined 6 patients with a 19mm electrode and 9 cases with a 17mm electrode and found a negative correlation between the insertion depth angle and the A value for the 17mm electrode, indicating that larger diameter cochleae had smaller insertion angles. Comparing these results to our study is difficult because Escude's study used different electrode lengths and used perimodiolar electrodes. Franke-Trieger found a significant correlation between insertion angle and cochlear size but this study involved sequential insertions of progressively longer electrodes into a temporal bone, potentially dilating the scala tympani. Perhaps a simple anatomical relationship does not exist between whereby a larger cochlear duct length results in greater degrees of insertion. Instead, other cochlear metrics such as cochlear height or the degree of curvature between turns are more important [29].

Our study found no statistical correlation between the degree of insertion and the postoperative outcomes at 6 months with both the length electrodes. This is consistent with several previous studies [30–33]. Spiral ganglion neurons do not extend for the full distance of the organ or Corti but rather end after approximately 720 degrees of rotation thereby potentially limiting any adverse effect of shallower insertions [34].

### 4.1. Correlations between Cochlear Size and Speech Outcomes

There are limitations of comparing the 31mm with the 28mm electrode for different sized cochlea. Ideally one should compare the same sized cochlea and compare the performance between the two different lengths electrodes that are inserted in the same manner. However, practically, it would be difficult to find two exactly similar sized cochleae in all dimensions including length, height, and width as well as with the same level of hearing loss and spiral ganglion distribution. There are also other factors which cannot be controlled for such as incomplete insertion. Surrogates have to therefore be utilised and in this case we have chosen to choose age, hearing loss, and a well-established cochlear metric.

When each individual electrode group was analysed separately, larger cochlear sizes were associated with better speech performance for the Flex 28 group. When the Flex 31 electrode was used, there did not appear to be any correlations between cochlea size and speech performance. What is interesting is that this association is electrode specific and needs to be reconciled with the observation that insertion depth and speech perception are not well correlated. The reasons for this are unclear and need to be explored in future studies. A shorter electrode may be less traumatic in a relatively longer cochlea, leading to a less traumatic insertion. A larger cochlea may also have a different distribution of spiral ganglion neurons which may be more conducive to stimulation by a shorter electrode. A smaller electrode in a larger cochlea may also lead to a lower risk of postoperative electrode migration, a factor poorly studied in the literature. As this is a retrospective study, this finding does not necessarily indicate that one should choose a shorter electrode for a relatively longer cochlea. What this does indicate is that cochlear size may be an important factor in determining CI outcomes. Attempts to predict the appropriate electrode length relative to cochlear size need to be examined to determine the effectiveness of such a technique [18].

A study by Johnston et al. is very similar to ours and compared retrospectively the outcomes of Flex 28 and Flex 31 electrodes [35]. Early postoperative outcomes at 3 months were assessed and postoperative X-rays were used as in our study. They similarly found early speech outcomes were not associated with electrode length or insertion depth. However, they did find that only in the Flex 28 group patients with incomplete insertions had shorter cochlear ducts lengths. Whilst we did not examine the degree of incomplete insertions (either on X-ray which can be difficult, or by the number of deactivated electrodes), it does suggest the possibility that longer cochlear duct lengths result in more complete insertions and therefore better utilization of the electrode contacts.

## 5. Conclusions

Cochlear metrics can be measured using routinely available radiological software using the preoperative temporal bone CT scan. The size of the cochlea appears to be an important factor which may affect CI outcomes for certain electrode lengths. This factor should be considered in future strategies for electrode selection.

## Figures and Tables

**Table 1:** Preoperative demographics. SD = standard deviation. P-value obtained using the Mann-Whitney U test and Chi square test.

Group	n	Mean age of implantation Years (SD)	Sex M: F	Side of
Flex 31	34	62 (14.5)	20:14	16 Left
				18 Right

Flex 28	21	63 (9.8)	10:11	8 Left
				13 Right

Combined	55	62 (12.8)	30: 25	24 Left
				31 Right

p-value		0.81	0.58	0.58

**Table 2:** Hearing and insertion outcome measures for each group. PTA = pure tone average at 250Hz, 500Hz and 1000Hz, HINT = hearing in noise test, and CNC = consonant nucleus consonant. P-value obtained using the Mann-Whitney U test, with significant values indicated in italic. Numbers in brackets indicate standard deviations.

	Flex 31	Flex 28	p-value
Preo op			
PTA (dB)	69 (13.2)	72 (8.9)	0.555
Best aided CNC quiet (%)	12.3 (15.8)	30.9 (16.1)	*0.0002*
HINT sentence quiet (%)	36 (24.9)	45.2 (22.2)	0.218
AZBIO sentence quiet (%)	n/a	33.3 (20.6)	
6 months post op			
Best aided CNC quiet (%)	52.6 (22.8)	59.7 (17.9)	0.399
HINT sentence quiet (%)	83.7 (21.8)	n/a	
AZBIO sentence quiet (%)	n/a	63.3 (19.99)	
CNC score shift (%)	40.7 (24.1)	28.9 (17.4)	0.055
Insertion outcomes			
(1) Degrees of electrode insertion on plain X-ray	489.4 degrees (82.47)	525 degrees (75.75)	0.165
(2) Number of channels inserted on plain X-ray (out of 12)	10.85 (1.08)	11.33 (0.73)	0.12
Correlation	*∗R=0.589*	*∗R=0.705*	
between (1) and (2)	(p<0.01)	(p<0.01)	

**Table 3:** Cochlear metrics for all subjects. There is no statistically significant difference in all measurements between subjects receiving either electrode.

		Minimum (mm)	Maximum (mm)	Mean (mm)	Standard Deviation (mm)
A-vaue	Flex 31	8.1	9.8	8.94	1.63
	Flex 28	8.1	9.5	8.87	0.38
	Combined			8.91	0.37

Outer wall length to 360 degrees	Flex 31	29.8	35.9	32.52	1.41
	Flex 28	27.8	34.7	31.91	1.63
	Combined			32.29	1.51

Outer wall length to 720 degrees	Flex 31	19.6	23.5	21.4	0.94
	Flex 28	18.3	22.6	21.1	1.09
	Combined			21.3	1

**Table 4:** A-value correlations with *postoperative* outcomes at 6 months. There are no statistically significant correlations between cochlear metrics listed on the left column and shifts in PTA, CNC, or AZBio scores which are not shown here. *∗∗* p<0.01, *∗* p<0.05.

	Flex 31		Flex 28		
	CNC (%)	HINT (%)	CNC (%)	AZBIO (%)	Electrodes inserted
Ac	0.36	0.34	*0.64∗∗*	*0.46∗*	0.19
Outer wall length 720^0^	0.19	0.19	*0.71∗∗*	*0.47∗*	0.27
Degree of insertion	0.23	0.22	-0.08	-0.09	
Electrodes inserted on X-ray	0.26	0.24	0.054	-0.08	

## Data Availability

The data used to support the findings of this study are restricted by the Sunnybrook Human Ethics Research Committee in order to protect patient privacy. Data are available from Dr. Jafri Kuthubutheen for researchers who meet the criteria for access to confidential data.
