# Corrigendum to “The Effect of Cochlear Size on Cochlear Implantation Outcomes”

**DOI:** 10.1155/2020/6407456

**Published:** 2020-10-01

**Authors:** Jafri Kuthubutheen, Amandeep Grewal, Sean Symons, Julian Nedzelski, David Shipp, Vincent Lin, Joseph Chen

**Affiliations:** ^1^School of Surgery, University of Western Australia, Crawley, Perth, Western Australia, Australia; ^2^Sunnybrook Health Sciences Centre, Toronto, Canada M4N 3M5; ^3^University of Toronto, Toronto, Canada M5S 1A1

In the article titled “The Effect of Cochlear Size on Cochlear Implantation Outcomes” [1], there was an error in Table 3, where there results following “Outer wall length to 360 degrees” and “Outer wall length to 720 degrees” were inverted. There was also a typo “*A*-vaue” which should have read “*A*-value”. The corrected table is shown below and is listed as Table 1.

## Figures and Tables

**Table 1:** Cochlear metrics for all subjects. There is no statistically significant difference in all measurements between subjects receiving either electrode.

		Minimum (mm)	Maximum (mm)	Mean (mm)	Standard deviation (mm)
*A*-value	Flex 31	8.1	9.8	8.94	1.63
	Flex 28	8.1	9.5	8.87	0.38
	Combined			8.91	0.37
Outer wall length to 720 degrees	Flex 31	29.8	35.9	32.52	1.41
	Flex 28	27.8	34.7	31.91	1.63
	Combined			32.29	1.51
Outer wall length to 360 degrees	Flex 31	19.6	23.5	21.4	0.94
	Flex 28	18.3	22.6	21.1	1.09
	Combined			21.3	1
